# Antibacterial Peptides Produced by Alcalase from Cowpea Seed Proteins

**DOI:** 10.3390/antibiotics10070870

**Published:** 2021-07-17

**Authors:** Ali Osman, Gamal Enan, Abdul-Raouf Al-Mohammadi, Seham Abdel-Shafi, Samar Abdel-Hameid, Mahmoud Z. Sitohy, Nashwa El-Gazzar

**Affiliations:** 1 Biochemistry Department, Faculty of Agriculture, Zagazig University, Zagazig 44511, Egypt; aokhalil@zu.edu.eg (A.O.); mzsitohy@hotmail.com (M.Z.S.); 2 Botany and Microbiology Department, Faculty of Science, Zagazig University, Zagazig44519, Egypt; hegazyseham@yahoo.com (S.A.-S.); dr.samar19891989@gmail.com (S.A.-H.); mora_sola1212@yahoo.com (N.E.-G.); 3 Department of Science, King Khalid Military Academy, Riyadh 11495, Saudi Arabia; almohammadi26@hotmail.com

**Keywords:** cowpea seed proteins, antibacterial activity, alcalase, protein hydrolysates, transmission electron microscopy, mass-spectrometry

## Abstract

Cowpea seed protein hydrolysates (CPH) were output from cowpea seeds applying alcalase^®^ from *Bacillus licheniformis*. CPH with an elevated level of hydrolysis was fractionated by size exclusion chromatography (SEC). Both CPH and SEC-portions showed to contain antimicrobial peptides (AMPs) as they inhibited both Gram-positive bacteria, such as *Listeria monocytogenes* LMG10470 *(L. monocytogenes)*, *Listeria innocua*. LMG11387 *(L. innocua*), *Staphylococcus aureus* ATCC25923 *(S.aureus*), and *Streptococcus pyogenes* ATCC19615 *(St.pyogenes*), and Gram-negative bacteria, such as *Klebsiella pnemoniae* ATCC43816 (*K. pnemoniae*), *Pseudomonas aeroginosa* ATCC26853 (*P. aeroginosa), Escherichia coli* ATCC25468) (*E.coli*) and *Salmonella typhimurium* ATCC14028 *(S. typhimurium*).The data exhibited that both CPH and size exclusion chromatography-fraction 1 (SEC-F1) showed high antibacterial efficiency versus almost all the assessed bacteria. The MIC of the AMPs within SEC-F1 and CPHs were (25 µg/mL) against *P. aeruginosa*, *E.coli* and *St. pyogenes*. However, higher MICsof approximately 100–150 µg/mL showed for both CPHs and SEC-F1 against both *S. aureus* and *L. innocua*; it was 50 µg/mL of CPH against *S.aureus*. The Electro-spray-ionization-mass-spectrometry (ESI-MS) of fraction (1) revealed 10 dipeptides with a molecular masses arranged from 184 Da to 364 Da and one Penta peptide with a molecular mass of approximately 659 Da inthe case of positive ions. While the negative ions showed 4 dipeptides with the molecular masses that arranged from 330 Da to 373 Da. Transmission electron microscope (TEM) demonstrated that the SEC-F1 induced changes in the bacterial cells affected. Thus, the results suggested that the hydrolysis of cowpea seed proteins by Alcalase is an uncomplicated appliance to intensify its antibacterial efficiency.

## 1. Introduction

Nowadays, there is long-term use and misuse of conventional antibiotics and consequently, bacterial drug resistance is developed and leads to a severe health issue worldwide [1,2,3,4,5,6]. Asthe discovery of other novel antibiotics is difficult, recent perspectives are challenged to find out an innovative way to inhibit multi drug-resistant microbial variants including the use of natural peptide [7], probiotics [8], natural plant extracts either singly or in combination with antibiotics [9,10,11], nanoparticles [12,13],and phage therapy [14].Therefore, it is not surprising that the World Health Organization (WHO) ranked antibiotic resistance as a priority disease encouraging the development of novel antibiotics [10,11,12,13].The best choice and most promising candidates are still cationic antimicrobial peptides or proteins (AMPs) [15].

In a previous study, a protein extracted from the cowpea bean [15] and other legumes, such as soybeans [16,17,18], chickpeas [19], etc. has been reported to produce significant antimicrobial action. AMPs possess clear advantages over the classical antibiotics as they were used successfully for food preservation [9] without the emergence of multidrug resistant bacteria variants; they also showed a promising modulation of the host immune response [2,20]. AMPs are a novel and recent alternative to classical antibiotics that possess a wide inhibitory spectrum against both Gram-positive and Gram-negative pathogenic bacteria with little or no capacity to induce antimicrobial resistance [21]. This showed a further search for obtaining AMPs with broad antimicrobial activity.

A variety of methods have been proposed to enhance protein antibacterial efficacy, including chemical modifications, such as esterification [22,23,24,25,26] and enzymatic hydrolysis by different enzymes [27,28,29,30].Cowpea (*Vigna unguiculata*) is a major crop of legumes globally. In both human and animal diets, it serves as a main dietary protein source and its protein content makes it a good raw material for obtaining protein extracts and hydrolysates [31].Cowpea bean seed proteins range from 22% to 30% protein in a dry basis. The kind of enzyme utilized in bioactive peptide preparations defines hydrolysate properties and the peptides that can be separated [32]. In previous studies, successive enzymatic systems, such as Alcalase^®^-Flavourzyme^®^ (AF) and pepsin-pancreatin (PP), were used for the generating of hydrolysates and peptides which had useful effects on antidiabetics [31,32,33,34,35], antihypertension [32], hypocholesterolemia activity [36], antioxidant activity [37], and functional properties [7,38].

Biological activities depend on the structure and conformation of proteins. Proteolysis, besides decreasing themolecular weight, also increases the number of ionizablegroups and can expose hydrophobic groups, which can change physical and environmental interactions. Alcalase^®^ enzyme extracted from *Bacillus licheniformis* [39]contains various proteinases with much specificity. Alcalase has been applied globally to present soluble hydrolysates of milk protein [25]and bioactive peptides extracted from fish [40]. At present, the antibacterial efficiency of CPH obtained by the action of alcalase has not been assayed. Therefore, the aim of the present work was to study the antibacterial activity of the obtained CPH by alcalase and fractions made by the size exclusion chromatography (SEC) technique and their action in bacterial cells using TEM studies.

## 2. Results

### 2.1. Production of Both CPH and SEC-Fractions 

**Figure 1:**
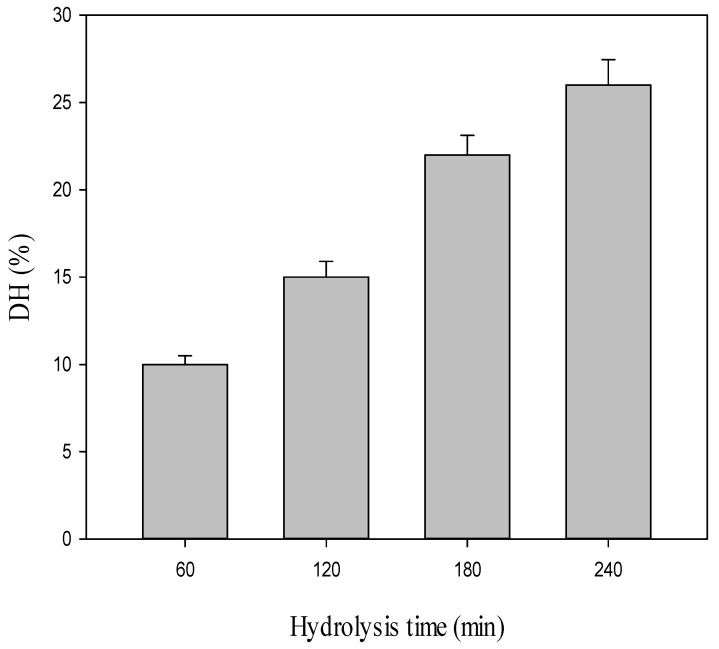
Degree of hydrolysis percentage (DH%) of cowpea protein hydrolysate (CPH) obtained by treatment with Alcalase (E/substrate ratio 1:200) for 240 min at 55°C and pH 7.8.

Cowpea protein isolate (CPI) is a good source of protein (92% protein) to be applied as a starting substrate for enzymatic proteolysis. CPI was hydrolyzed with Alcalase (enzyme/substrate ratio 1:200) at 55°C and pH 7.8 with varied proteolysis times from 60 to 240 min. The degree of hydrolysis (DH) was estimated for the obtained hydrolysates with this treatment and the data are given in Figure 1. CPH obtained after 60, 120, 180 and 240 min showed DH rates of 10%, 15%, 22% and 26%, respectively.

**Figure 2:**
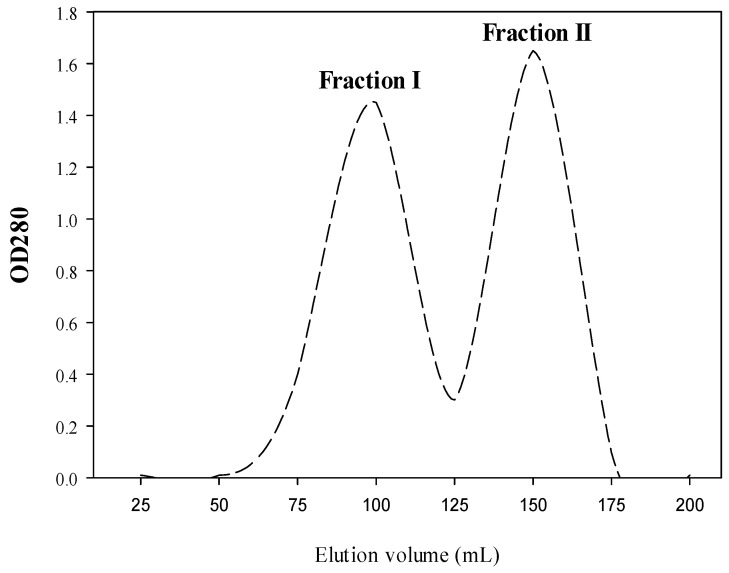
Size exclusion chromatography (SEC) using Sephadex G-25 of the 4 h cowpea seed protein hydrolysate (CPH) obtained by treatment with Alcalase.

The antibacterial activity of CPH was estimated. CPH with a DH of approximately 26%, generated by treatment with Alcalase for 240 min, gave the highest inhibitory activity against Gram-positive and Gram-negative bacteria. This was purified by gel filtration on Sephadex G-25 to produce the bioactive fractions. As reported in Figure 2, there are two major absorbance peaks (fraction 1 and fraction 2) at 280 nm (Figure 2). Fractions correlated with each peak were dialyzed against distilled water for 3 days, lyophilized, and then estimated for their antibacterial activity.

### 2.2. Electro-Spray-Ionization-Mass-Spectrometry (ESI-MS)of SEC-F1

**Figure 3:**
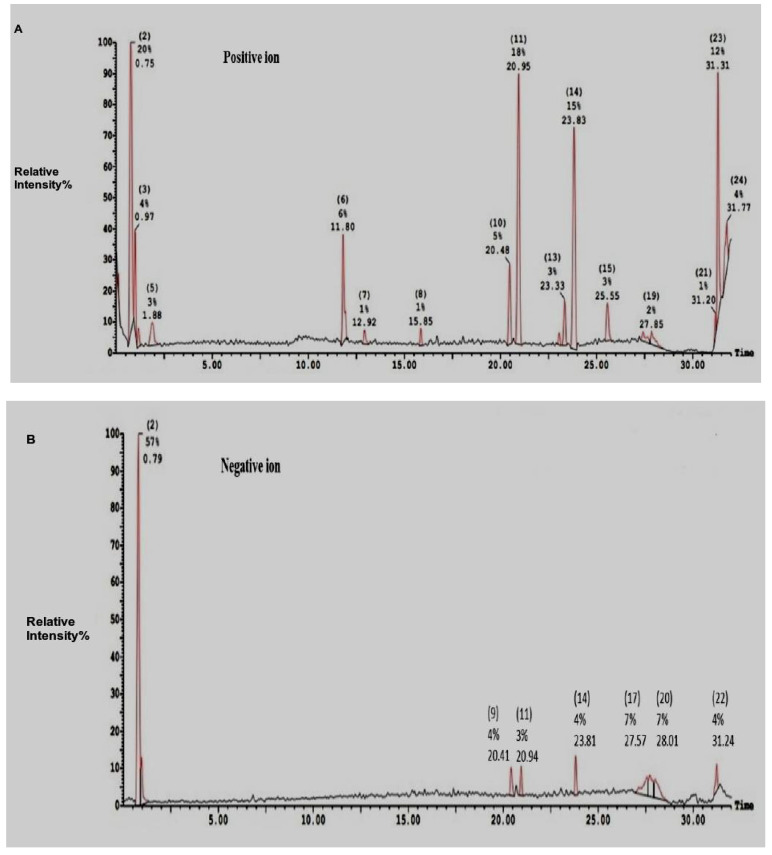
Chromatogram of peptides formation from size exclusion chromatography of fraction 1 showing (**A**): Positive ions portion and (**B**): Negative ions portion.

Fraction 1 obtained from gel filtration chromatography (Figure 2) displayed the highest antibacterial activity against the tested bacteria. Therefore, the AMPs in fraction 1 were analyzed by electro-spray-ionization-MS (ESI-MS) and included both positive and negative ions for molecular weight determination; the identification of the AMPsand the main peaks are given in Figure 3.

The possible peptide compositions of fraction (1) estimated by ESI-MS are given in Table 1. The AMPs within SEC-F1 were investigated by Electro-Spray-Ionization-MS. This was mandatory to assess the bioactivity of components within the AMPs. Results are given in Table 1. The positive ions fraction elucidated 9-dipeptides with molecular masses ranging from 184.94 Da to 364 Da and one penta-peptide containing 5 amino acid residues, such asCya am, Trp, Met and Arg. In view of the amino acid composition of the positive ions fraction, non-polar hydrophobic amino acids, such as glycine, alanine, valine, leucine, and tryptophan, predominated the composition of these bioactive peptides. Regarding the negative ion-fractions, peptides of molecular masses in the range 318 Da to 373 Da were shown and included 4-dipeptides viz. Trp-Cys am, Trp-Trp, Arg-Cys am and Phe-Trp. Also, a tetra-peptide was shown viz. Trp-Met-Arg-Cys am (Table 1). In view of the amino acids within the negative ions fraction, the majority of amino acids were hydrophobic, e.g. tryptophan (5residues), methionine (1 residue), Phenylalanine (1 residue). Three peptides out of the 10 peptides revealed in positive ions mode contained arginine, i.e. they are cationic peptides with positive charges. Likewise two peptides out of the five peptides revealed in the negative ions mode contained arginine, i.e. they are cationic peptides. Both hydrophobic and alkaline peptides are known for their antimicrobial activity [40]. 

### 2.3. Antibacterial Activity 

The CPH (DH, 26%) and its two fractions obtained by gel filtration chromatography (Figure 2) were bioassayed against Gram-positive and Gram-negative bacteria. The antibiotic ciprofloxacin was used as a positive control. The results are given in Table 2. The AMPs of both CPH and SEC-F1 showed distinctive antibacterial activity against the indicator organisms tested than that obtained by SEC-F2. This inhibitory activity of the AMPs (CPHs, SEC-F1) against the sensitive bacteria matched almost that obtained by the antibiotic ciprofloxacin (10µg/mL), except for *S.aureus,* which showed more inhibition by the AMPs than that obtained by the antibiotic ciprofloxacin. Almost all the indicator bacteria were significantly inhibited by both CPH and SEC-F1.The diameters of the inhibition zones were in the range 16–26mm (Table 2). Consequently, both CPH and SEC-F1 were used for further studies.

### 2.4. Minimum Inhibitory Concentration (MIC) of Both CPHs and SEC-Fractions 

The MIC values of both CPHs and SEC-F1 were 25 µg/mL versus *S. typhimurium*, *K. pneumoniae*, *St. pyogenes*, *L. monocytogenes*, *P. aeruginosa* and *E. coli* (Table 3). They were of approximately 100 µg/mL of SEC-F1 and of approximately 150 µg/mL; 50 µg/mL of CPHs against *L. innocua, S. aureus,* respectively (Table 3). The MIC of the control antibiotic ciprofloxacin was shown to be 20 µg/mL.

### 2.5. Transmission Electron Microscope (TEM) of SEC-F1

**Figure 4:**
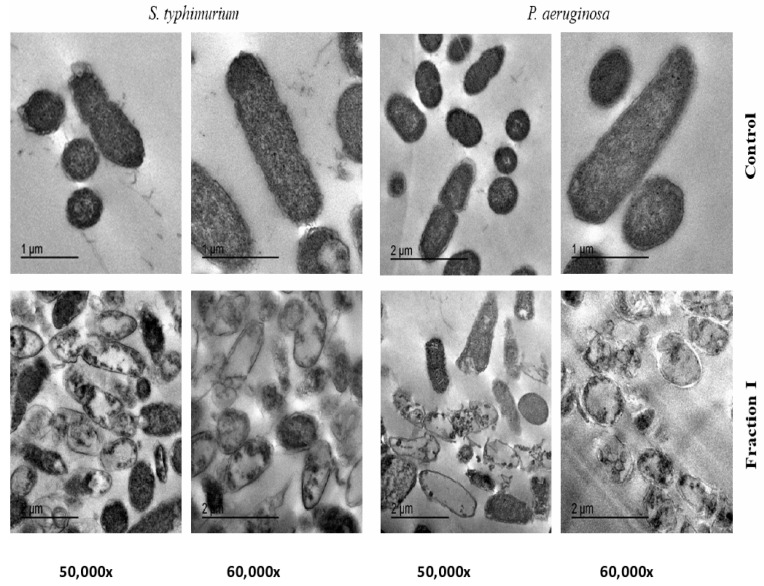
**T**ransmission electron microscope (TEM) of control and treated bacteria (*S. typhimurium* and *P. aeruginosa*) as affected by 25 µg/mL of size exclusion chromatography fraction number 1.

The treatment of both *S. typhimurium* and *P. aeruginosa* suspensions with 25µg/mL of SEC-F1 has led to an obvious increase in damaged cells after their incubation at 37°C, as exhibited by TEM images in Figure 4. The analysis of TEM images indicate that the cationic antimicrobial proteins cause overall deterioration of cell membranes, cell swelling, vacuoles composition and finally whole lysis of cell components.

## 3. Discussion

The high incidence of resistant bacteria variants to antibiotics has a vast impact on human mortality and healthcare [5,15,35]. Many bacteria have become resistant against many antimicrobial agents. Thus, there is an urgent demand to find other alternative antimicrobial agents which could kill the multidrug-resistant bacteria [15]. AMPs are highly active against most microbes, including both Gram-positive and Gram-negative bacteria [40].

AMPs are promising new antibacterial agents due to their killing mechanism via interaction with bacterial cell walls and membranes [19,41].They could be generated by different methods, such as chemical modification [23], microbial fermentation [42] and enzymatic proteolysis [29]. Alcalase has been used to generate biologically active peptides from different sources, such as barbel muscle protein [40], goat whey protein [27], sorghum protein [43], chickpea protein [44], canola protein [45], and egg [46],that cleaves the high molecular weight, releasing more active subunits with smaller molecular sizesand with a hydrophobic nature that showed greater antimicrobial activity [41]. This optimization produces effective bactericidal peptides that may be identified as potential antimicrobials [41,47,48].

Protein substrates play an important role in the biological activities of protein hydrolysates. In the current research, ocwpea protein isolate was used as a starting substrate for hydrolysis with Alcalase. A similar tendency of DH was determined from the hydrolysis of soy protein with Alcalase. [49]. The degree of hydrolysis was recorded for the obtained hydrolysates at different times, similar to that reported by Osman et al. [27]. The form of the hydrolysis curve in the present work is typical of those already published by Osman et al. [27]. It was reported from the previous studies that high DH by Alcalase is necessary for the most active protein solubility, emulsifying efficiency and adequate functionality. The solubility of cowpea protein hydrolysate was adequately optimized by response surface techniques, and the hydrolysate recorded a potent functionality [50].

The antibacterial activity of CPH at different times (60–240 min) was estimated. CPH, with a DH of 26% generated by treatment with Alcalase for 240 min presented the highest action against Gram-positive and Gram-negative bacteria. This antibacterial efficiency showed greater inhibitory zones against *almost all the tested bacteria*. This is probably due to the liberation of some antibacterial peptides upon Alcalase hydrolysis of the cowpea seed proteins [28]. The interaction of antimicrobial peptides with bacterial cells is dependent on their amino acid composition and their methyl groups that process cationic charges which could connect to cell membrane bilayers, and in turn cause pore formation [41]. This mechanism might happen through electrostatic binding between the positively charged parts of the cationic proteins and the negatively charged layers of the cell membrane rising from teichoic acid and phospholipid components causing cell degeneration, and in turn cell lysis or destructionleading to leakage of cell electrolytes [51].

Due to the high antibacterial activity of CPH, it was purified by gel filtration onSephadex G-25 to produce the bioactive fractions. Concerning the bioactive components within the investigated AMPs, the positive ions portion include non-polar hydrophobic amino acids in 9 out of 11 residues investigated, such as Cys am-Trp, Cys am-Trp, Trp-Ser, His-Cys am, Trp-Met-Arg (penta-peptide), Trp-Cys am (2 residues), Leu-Ala, Asn-Val and Arg-Gly, which were known to attach the negatively charged phospholipid of bacterial cell membranes; making electrostatic forces which could make pores within bacterial cell membranes; from which cell electrolytes can emerges outside bacterial cells [15,22]. Also, the residues containing Sulphur, such as met within the pentapeptide and cysteine showed to be of distinctive antimicrobial activity [52]. The bioactive components within the amino acids of the negative ions portion contained tryptophane within 4 residues out of 5 residues detected and showed to be bioactive components, as shown above, for the positive ions of fraction [41]. Moreover, the releasing of peptides with a smaller molecular mass by alcalase is of more mobility and inhibitory activity than the ones with high molecular weight [53]. In addition, the Arg-Cys am residue showed previouslya distinctive bioactivity against bacteria since the amino group of arginine could accept the proton-giving, positively-charged NH3^+^ group, which could attach with the negatively- charged bacterial cell membrane, giving bacterial death [52].

The recent perspectives are to use the AMPs in mixtures as these mixtures showed greater antimicrobial activity. Pfalzgraff et al. [54] discussed the antimicrobial activity of some AMPs and their therapeutic potential for skin infections and wounds. The AMPs in a mixed peptide showed promising use as a surface therapy and inhibited skin infections caused by *Enterococcus faecium, Staphylococcus aureus, Pseudomonas aeruginosa, Klebsiella pneumonia* and *Acenetobacter baumanni.*

The effects of SEC-F1 peptides that appeared herein showed several signs of cellular deformation as shown by TEM- studies, reflecting a direct disruptive influence of this fraction on the cell wall and cell membrane. Distorted cells pointed to cell shrinkage, cell membrane wrinkles, and pore formation, and also some emptiness of cellular live material [45]. These results are in confirmation with the previous reports of the direct interaction of cationic antimicrobial peptides with the cell membranes and follow previously published works [41,48].

Thus, the results provide critical information on CPH that may be used as active ingredients to formulate antibacterial peptides. Further work will be necessary to improve the inhibitory activity of AMPs by their chemical modification via their methylation, and to investigate the antimicrobial activity of such peptides in foods. The antimicrobial activity of these peptides in combination with antibiotics is also necessary. Work in this respect is in progress.

## 4. Materials and Methods

### 4.1. Collection of Pathogenic Bacteria

Both Gram-positive bacteria, such as *L. monocytogenes, L. innocua*, *S. aureus*, and *St. pyogenes*, and Gram-negative bacteria, such as *K. pnemoniae*, *P. aeroginosa, E. coli* and *S. typhimurium* were used. All the bacteria were stored as stock cultures in glass beads at−20 °C, subcultured and propagated in a brain heart infusion broth (Oxoid). Prior to the microbiological work, slope cultures were grown onto the brain heart infusion broth (Oxoid) as described previously by Abdel-Shafi et al., 2019 [15] and stored at 4°C during experiments.

### 4.2. Plant Materials and Chemicals

The cowpea (*Vigna unguiculata*, L.) seeds were provided from local market, Zagazig, Sharkia Governorate, Egypt. These seeds were identified by Dr. Samir Salem, Botany, Dept. Fa of Sci., Za. Univ, Egypt. Alcalase enzyme is a product metabolite from *B. licheniformis;* it was purchased from Sigma (St. Louis, MO, USA).

### 4.3. Cowpea Protein Isolation

Cowpea seeds (1 kg) were ground and n-hexane (5% w/v) was used for 8 h to produce flour. On a rotary evaporator, the solvent was evaporated and the dried-defatted meal was deposited at 4°C before analysis was performed. The total protein was isolated from the defatted flour by precipitation with 0.1 N HCl at pH 4.5 using the procedures previously mentioned [16,22], dialyzed against distilled water for 4 days, lyophilized, and stored at−20 °C until used.

### 4.4. Cowpea Seed Protein Hydrolysates Preparation

The CPHs were prepared as described by Otte et al. [55]. In 100 mL of distilled water, lyophilized cowpea protein isolate (10 g) was dissolved and the pH was adjusted to 7.8. In order to achieve a final enzyme/substrate (E/S) ratio of 1:200, Alcalase was added. The reaction was permitted to continue for 240 min under continuous stirring at 55 °C, with the pH held at 7.8. The reaction was prevented by heating for 10 min at 100 °C and then cooling at 4 °C in an ice bath. The collected protein hydrolysates were freeze-dried and stored at −4°C. The degree of hydrolysis (DH) was calculated every 60 min during hydrolysis according to Adler-Nissen [56]. The hydrolysate was clarified by centrifugation at 4000× *g* for 30 min at 16 °C to remove insoluble substrate fragments, and the supernatant was lyophilized and kept frozen at −20 °C until used. The antibacterial activity of the CPH was tested against the pathogenic bacteria used and the highest antibacterial agent was separated by sizeexclusion chromatography (SEC).

### 4.5. Fractionation of CPH by Size Exclusion Chromatography (SEC) 

The CPH obtained after 240 min of hydrolysis time with Alcalase, with the highest antibacterial activity and 26% degree of hydrolysis was fractionated by SEC using a Sephadex G-25 gel filtration column (1.6 × 150 cm). After dissolving the CPH in deionized water, 10mL of the CPH solution were added to the column and eluted with distilled water at a flow rate of 1 mL/min, and the OD was measured at 280 nm. The main peaks were collected and lyophilized to evaluate their antibacterial efficiency.

### 4.6. Electro-Spray-Ionization-Mass-Spectrometry (ESI-MS) of SEC-F1

The main peak (SEC-F1) with the highest antibacterial activity was subjected to electro-spray-ionization-mass-spectrometry (ESI-MS) positive and negative ion. An aliquot of approximately 10 µl of the final peptide solution was injected into the chromatograph and the peptides were separated on a XEVO TQD triple quadruple instrument Water Corporation, Milford, MA01757 U.S.A, mass spectrometer. Column: ACQUITY UPLC-BEH C18 1.7 µm−2.1 × 50 mm Column with flow rate: 0.2 mL\min using solvent system: consisted of (A) Water containing 0.1% formic acid (B) Acetonitrile containing 0.1% formic acid [41].

### 4.7. Antibacterial Activityof CPH and Fractions Obtained by SEC

Both CPH and SEC fractions (F1 and F2) were bio assayed against Gram-positive bacteria, such as *L. monocytogenes*, *L. innocua*, *S. aureus* and *St. pyogenes*, and Gram-negative bacteria, such as *K. pneumonia*, *P. aeruginosa*, *E. coli* and *S. typhimurium* by the Kirby-Bauer disk-diffusion method [57]. The indicator bacteria were swabbed onto the surface of brain heart infusion agar (Oxoid) plates. Then, filter paper discs were soaked in either CPH or SEC fractions for 15 min and put onto the agar plates that were previously seeded with the indicator bacteria. After incubation for 24 h, diameters of inhibition zones were measured by anmm ruler after subtracting the diameter of the filter paper disc [48,57,58].

### 4.8. Minimum Inhibitory Concentration (MIC) Determination of Both CPH and SEC-F1

MIC values of both CPH and SEC-F1 were tested against both Gram-positive bacteria (*L. monocytogenes, L.* *innocua, S. aureus* and *St. pyogenes*) and Gram-negative bacteria (*K. pneumoniae, P. aeruginosa, E. coli* and *S. typhi*) by the Kirby-Bauer disk-diffusion method. The bacterial suspension was swabbed onto the surface of the brain heart infusion agar (Oxoid) plates. Then filter paper disks of 6mm in diameter were soaked in either CPH or SEC F1 (25, 50, 100, 250, 500, 1000 μg/mL) and placed onto an agar surface with suitable distances separating them from each other. The plates were incubated at 37°C for 24 h and inhibition zone diameters (mm) were measured through a millimeter ruler [59].

### 4.9. Transmission Electron Microscope (TEM) of SEC-F1

Since, both *S. typhimurium* and *P. aeruginosa* were highly inhibited by either CPH or SEC-F1 AMPs, they were selected for TEM studies. They were also chosen as the authors investigating the action of these peptides in later work [15]. *S. typhi* and *P. aeruginosa* were selected for TEM. The bacteria were grown in a brain heart infusion broth and incubated at 37 °C to record a growth of approximately 10^9^ CFU/ mL, which was consequently diluted to the desired CFU mL^−1^. The MICs aliquots (25 μgmL^−1^ of SEC-F1) were added to both the *S. typhi* and *P. aeruginosa* cell suspensions, respectively, except control, and incubated at 37 °C for 4 h. Ultrathin sections were prepared for study by TEM. Perfusion or immersion fixation of the bacteria was determined through the method adopted by Morris [60]. The cells were kept overnight at 4°C, then washed 3 times for 15 min in a 0.1 M sodium phosphate buffer + 0.1 M sucrose and post fixed 90 min using a 2% Na phosphate buffered osmium tetroxide (pH 7.4). They were then washed 3× for 15 min in a 0.1 M sodium phosphate buffer pH 7.4 and dehydrated2×for 15 min: 50% ethanol (in distilled water). They were then contrasted overnight using 70% acetone + 0.5% uranyl acetate + 1% phosphor tungstic acid at 4°C, 2× for 15 min, 80% ethanol, 2×for 15 min, 90% ethanol, 2× for 15 min, 96% ethanol, 3× for 20 min, and 100% ethanol,2×for 15 min. Then 30 min. 2:1 acetone: Epon mixture, 30 min 1:1 acetone: Epon mixture,30 min 1:2 acetone: Epon mixture, Epon pure solution overnight at 4°C and finally new fresh Epon solution. Consequently, they were placed in an incubator for 48 hat 65°C for polymerization, and were then cut with an ultra-microtome set to 50–100 nm section thickness. Then the sections were washed to grids made of copper or nickel. Post contrastation of the sections was determined 10 min 8% uranyl acetate and 5 min 0.7% lead citrate + 0.9% sodium citrate after drying for 15 min; sections were demonstrated in a TEM, by a JEOL 2100 TEM at 80 KV at EM Unit, Mansoura University, Egypt [61,62,63].

### 4.10. Statistical Analysis

All the experiments were conducted in triplicate and the results were expressed using one-way ANOVA analysis for estimating means and standard deviations (±SD) [64]. The test was followed by the least significant difference (LSD) test with statistical WASP software version 2.0; LSD, at significant level (*p*<0.05).Sample symbols (a.a): Mean non-significant difference; (a.b): Mean significant difference [61,65].

## 5. Conclusion

The CPHs were extracted from the *cowpea seeds and inhibited many pathogenic bacteria.* TheAMPswere provided by treatment of CPHs by the enzyme alcalase. The AMPs were characterized using ESI-MS Spectroscopy. The MICs of the AMPs were in the range 25–150 µg/mL against all the bacteria tested. The mode of action of the most active AMPs was studied by TEM studies against sensitive bacteria. 

## Figures and Tables

**Table 1:** Possible peptide compositions of AMPs within size exclusion chromatography fraction 1 (SEC-F1) estimated by electro-spray-ionization MS (ESI-MS) technique.

Ions Mode/Peaks Number		Area (%)	Molecular Weight	Composition
Positive ions	2	20.20	707.32 (364)	Cys am-Trp
	3	4.10	707.28 (364)	Cys am-Trp
	5	2.57	192.05	Ser-Cys
	6	6.46	274.20	Trp-Ser
	10	4.57	331.28	Cys-Arg
	11	18.28	659.43 (312 & 318)	His-Cysam & Trp-Met & Arg-Cys am
	13	2.54	359.31	Trp-Cys am
	14	15.29	359.31	Trp-Cys am
	23	12.09	184.94	Leu-Ala
			226.89	Gln-Pro
	24	3.86	214.06	Asn-Val; Arg-Gly
Negative ions	2	57.28	719.35 fragmented into (365)	Trp-Cys am
	9	3.94	373	Trp-Trp
	11	3.27	656.97 fragmented into (318)	Trp-Met & Arg-Cys am
	17	7.07	1133.01 fragmented into (330)	Arg-Cys am
	20	7.31	532.97 fragmented into (332)	Phe-Trp

**Table 2:** Antibacterial activity of cowpea protein hydrolysate (CPH) and size exclusion chromatography (SEC) fractions (1 and 2) against tested bacteria.

Bacteria		Inhibition Zone Diameter (mm)			
		Ciprofloxacin (100µg/mL)	CPH	SEC-Fraction 1	SEC-Fraction 2
*G +VeBacteria*	*St. pyogenes*	20.0 ± 0.19	24.70 a ± 0.51	26.50 a ± 0.67	8.50 b ± 0.31
	*L. monocytogens*	23.0 ± 0.45	22.30 a ± 0.47	21.50 b ± 0.34	00.00
	*L. innocua*	22.0 ± 0.47	21.50 b ± 0.67	22.60 a ± 0.67	7.60 c ± 0.27
	*S. aureus*	18.0 ± 0.22	26.30 a ± 0.34	23.50 b ± 0.66	00.00
*G-VeBacteria*	*S. typhimurium*	21.0 ± 0.12	24.30 a ± 0.31	21.63 b ± 0.12	9.00 c ± 0.11
	*k. pneumonia*	18.0 ± 0.21	20.80 b ± 0.19	21.50 a ± 0.81	8.300 c ± 0.17
	*P. auriginosa*	21.0 ± 0.12	25.30 a ± 0.12	18.3.50 b ± 0.22	9.30 c ± 0.61
	*E. coli*	18.0 ± 0.22	17.3.50 b ± 0.57	23.50 a ± 0.45	8.60 c ± 0.86

Mean in the same row having different letters are significantly different (*p* ≤ 0.05).

**Table 3:** Minimum inhibitory concentration (MIC) of CPH and (SEC-F1) against tested bacteria.

Microorganisms	Inhibition Zone Diameter (mm/µg mL^−1^)											
	25		50		100		250		500		1000	
	CPH	F_1_	CPH	F_1_	CPH	F_1_	CPH	F_1_	CPH	F_1_	CPH	F_1_
*S. typhimurum*	11.0d± 0.3	15.0c ± 0.1	17.0b ± 0.3	18.0b ± 0.5	18.0b ± 0.1	18.6b ± 0.4	21.0b ± 0.5	22.0b ± 0.1	21.0c ± 0.7	29.0a ± 0.6	22.0d ± 0.5	29.6a ± 0.5
*K. pneumoniae*	15.6b± 0.3	12.0d ± 0.1	15.7c ± 0.8	12.7c ± 0.2	16.0 c ± 0.3	14.0 c ± 0.7	20.0b ± 0.6	15.0d ± 0.9	21.0c ± 0.4	18.0d ± 0.2	24.0c ± 0.4	2.02c ± 0.3
*St. pyogenes*	13.0c± 0.7	24.0a ± 0.4	17.9b ± 0.8	26.0a ± 0.7	20.0a ± 0.7	28.0 a ± 0.1	24.0a ± 0.2	18.0c ± 0.3	26.0b ± 0.1	20.0c ± 0.2	28.3b ± 0.3	27.0b ± 0.4
*L.monocytogen*	9.0e± 0.1	9.0e ± 0.3	9.5d ± 0.2	10.3d ± 0.3	10.0d ± 0.8	13.0 c ± 0.8	23.0ab ± 0.1	15.3d ± 0.4	18.3d ± 02	25.0b ± 0.1	19.6e ± 0.8	20.3d ± 0.1
*L. innocua*	0.0	0.0	0.0	0.0	0	11.3d ± 0.2	14.0c ± 0.5	12.7 ± 0.3	20.3c ± 0.4	13.3f ± 0.5	22.0d ± 0.2	22.0c ± 0.1
*P. aeruginosa*	18.0a± 0.5	18.0b ± 0.3	19.0a ± 0.4	19.0b ± 0.2	19.3a ± 0.5	19.3b ± 0.4	23.0ab ± 0.6	23.0b ± 0.9	20.00c ± 3	20.0c ± 0.2	20.0e ± 0.5	16.7e ± 0.2
*S. aureus*	0.0	0.0	12.3 ± 0.4	0.0	9.0d ± 0.93	11.0d ± 0.7	13.0c ± 0.3	13.0e ± 0.2	22.0c ± 0.3	16.0e ± 0.5	31.0a ± 0.8	17.7e ± 0.4
*E. coli*	15.0b± 0.15	24.0a ± 0.3	20.6a ± 0.3	25.7a ± 0.1	19.3a ± 0.1	27.0a ± 0.5	26.0a ± 0.4	29.0a ± 0.1	30.0a ± 0.4	31.0a ± 0.4	32.0a ± 0.7	31.3a ± 0.3

Mean in the same row having different letters are significantly different (*p* ≤ 0.05).

## Data Availability

The data presented in this study are available in the article.
